# Bi-Directional Brillouin Optical Time Domain Analyzer System for Long Range Distributed Sensing

**DOI:** 10.3390/s16122156

**Published:** 2016-12-16

**Authors:** Nan Guo, Liang Wang, Jie Wang, Chao Jin, Hwa-Yaw Tam, A. Ping Zhang, Chao Lu

**Affiliations:** 1 Department of Electronic and Information Engineering, The Hong Kong Polytechnic University, Kowloon, Hong Kong, China; 13901767r@connect.polyu.hk (N.G.); 15901369r@connect.polyu.hk (C.J.); chao.lu@polyu.edu.hk (C.L.); 2 Department of Electronic Engineering, The Chinese University of Hong Kong, Shatin, Hong Kong, China; 3 Department of Electrical Engineering, The Hong Kong Polytechnic University, Kowloon, Hong Kong, China; jayjay.wang@connect.polyu.hk (J.W.); hwa-yaw.tam@polyu.edu.hk (H.-Y.T.); aping.zhang@polyu.edu.hk (A.P.Z.)

**Keywords:** fiber optics sensors, Brillouin scattering, temperature, optical time domain analyzer

## Abstract

We propose and experimentally demonstrate a novel scheme of bi-directional Brillouin time domain analyzer (BD-BOTDA) to extend the sensing range. By deploying two pump-probe pairs at two different wavelengths, the Brillouin frequency shift (BFS) distribution over each half of the whole fiber can be obtained with the simultaneous detection of Brillouin signals in both channels. Compared to the conventional unidirectional BOTDA system of the same sensing range, the proposed BD-BOTDA scheme enables distributed sensing with a performance level comparable to the conventional one with half of the sensing range and a spatial resolution of 2 m, while maintaining the Brillouin signal-to-noise ratio (SNR) and the BFS uncertainty. Based on this technique, we have achieved distributed temperature sensing with a measurement range of 81.9 km fiber at a spatial resolution of 2 m and BFS uncertainty of ~0.44 MHz without introducing any complicated components or schemes.

## 1. Introduction

Since the concept of the Brillouin time domain analyzer (BOTDA) was first proposed in 1989 [1], enormous research efforts have been devoted to this technique, due to its capability of distributed monitoring of temperature and strain change along the fiber. By scanning the pump-probe frequency difference, the Brillouin gain spectrum (BGS) can be re-constructed to determine the distribution of Brillouin frequency shift (BFS), and hence the temperature and/or strain information along the fiber can be retrieved. In recent decades, driven by the increasing demand for structural health monitoring by distributed temperature and/or strain sensing in civil, transportation, aviation, and military applications, many works have been done to enhance the system performance in terms of spatial resolution and sensing range [2]. However, it has been shown that it is difficult to extend the sensing range while maintaining a high spatial resolution, since the BOTDA system suffers from pump depletion [3], non-local effects [4], modulation instability [5], and other nonlinearities. Due to these limitations, the signal-to-noise ratio (SNR) of the Brillouin time-domain signal at the far end of the fiber is severely degraded, especially for long-range sensing at high spatial resolution, leading to inevitable large BFS uncertainties and temperature/strain error.

In recent years, to enhance the SNR of BOTDA systems, various techniques and schemes have been established [6,7,8,9,10,11,12,13,14,15,16,17,18,19]. Coherent detection has been applied for Brillouin signal detection to improve the SNR [7,8] and to obtain additional Brillouin phase information for reduction of the non-local effects [9]. Optical coding techniques have been employed to achieve highly sensitive BOTDA systems without sacrifice of the spatial resolution [10,11]. Moreover, in order to reduce the pump loss and depletion, distributed Raman and Brillouin amplification has been applied in BOTDA systems [12,13]. Further works have combined the coding technique with Raman amplification in BOTDAs to extend the sensing range with less system performance degradation [14,15,16]. More recently, different kinds of offline signal processing techniques such as image processing [17] and artificial neural networks [18] have been deployed to significantly enhance the sensor performance.

Recently, we have reported a novel scheme named Bi-Directional BOTDA (BD-BOTDA) system [19] to extend the sensing range of the system but without introducing any complicated components. In this paper, the BD-BOTDA system is further demonstrated with optimized parameters, achieving distributed sensing over 80 km at a spatial resolution of 2 m and a BFS uncertainty of ~0.44 MHz. Unlike the preliminary results in [19], here we also analyze the system performance and compare it with that of the conventional unidirectional BOTDA. The results show that, at the same sensing range (e.g., ~80 km), the BD-BOTDA system has a performance level comparable to the unidirectional BOTDA with half of the sensing range (e.g., ~40 km) and doubles the measurement speed. Moreover, the proposed technique shall provide an alternative approach to extending the sensing range. This technique does not compete with other existing techniques, such as Raman amplification [12], Brillouin amplification [13], and coding techniques. Thus, it is expected that the sensing range can be further enhanced by combination of our approach with other existing techniques.

## 2. Principle and Experimental Setup

**Figure 1:**
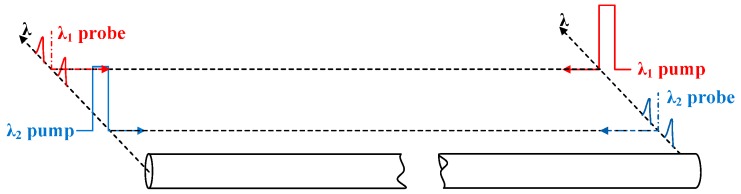
Principle of BD-BOTDA: two pump-probe pairs including pulsed pumps and CW probes at two wavelengths are injected into both ends of the FUT in opposite directions.

As shown in Figure 1, our BD-BOTDA system employs two pump-probe pairs whereby each pair has a particular wavelength. Each pump-probe pair has a pulsed pump and a continuous wave (CW) probe, which are each injected from opposite ends of the fiber under test (FUT) and serve as one of the two channels of our BD-BOTDA system. For each channel, one half of the whole fiber span is used as the sensing fiber, while the other half only carries the probe signal to the sensing fiber. The two channels simultaneously measure the Brillouin signals along each half of the FUT, and the temperature or strain information along the whole FUT can hence be obtained by combining the results collected from the two channels of the BD-BOTDA system. If the system parameters (e.g., pump and probe powers) involved in the Brillouin interaction in the sensing fiber are optimized for each channel, both the SNR performance and the measurement time of the BD-BOTDA system shall be the same as a unidirectional BOTDA system with half of the sensing range. That means the system performance becomes better while the measurement speed is doubled compared with the unidirectional BOTDA system of the same sensing range. Compared with the unidirectional BOTDA system, some extra components are necessary. However, a number of key components can be shared by the two wavelength channels.

**Figure 2:**
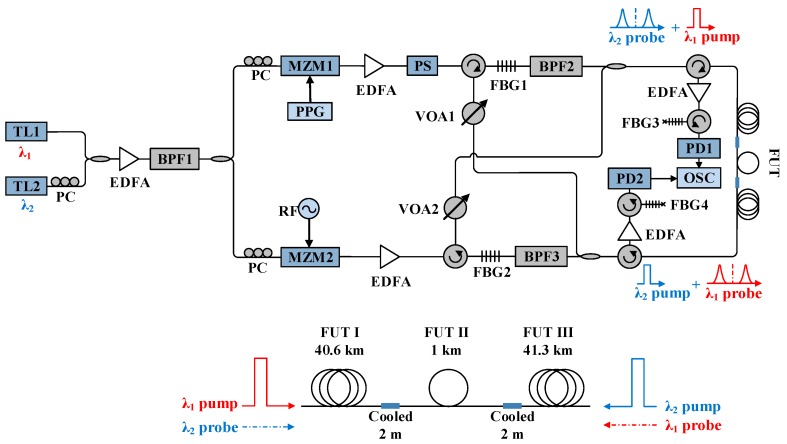
Experiment setup of BD-BOTDA system. TL: Tunable laser; PC: Polarization controller; EDFA: Erbium-doped fiber amplifier; BPF: Band pass filter; MZM: Mach–Zehnder modulator; PPG: Pulse pattern generator; PS: Polarization scrambler; VOA: Variable optical attenuator; FBG: Fiber Bragg grating; PD: Photodiode; OSC: Oscilloscope; FUT: Fiber under test.

The experiment setup for the BD-BOTDA system is illustrated in Figure 2. Two tunable lasers (TL1 and TL2, Emcore Corporation, Alhambra, CA, USA) with the wavelengths of *λ*_1_ (~1550 nm) and *λ*_2_ (~1552.8 nm) are employed for the two channels of BD-BOTDA, respectively. The output of TL2 passes through a polarization controller (PC) to make its polarization aligned with TL1 before they are coupled together with a 50/50 coupler. An erbium-doped fiber amplifier (EDFA, Amonics Ltd., Kowloon, Hong Kong, China) is used to boost the optical power, and the following band pass filter (BPF1) with a bandwidth of 5 nm is used to filter out the amplified spontaneous emission (ASE) noise. Then, the lightwaves are divided into two branches for the generation of two pairs of pulsed pump and CW probe. The upper branch is modulated by utilizing a Mach–Zehnder modulator (MZM1, iXBlue, Besançon, France) and a pulse pattern generator (PPG, Anritsu Corporation, Atsugi-shi, Kanagawa, Japan) to produce 20 ns pump pulses at both *λ*_1_ and *λ*_2_. The EDFA in the upper branch is used to boost the pulse peak power and the polarization scrambler (PS, General Photonics Corporation, Chino, CA, USA) is used to minimize the polarization-dependent fluctuation of the Brillouin signal. A circulator together with a fiber Bragg grating (FBG1) having a reflection band at *λ*_2_ is applied to separate the two channels of pump pulses. The following BPF2 with a bandwidth of 1 nm is employed to minimize the residual ASE noise and residual pulsed pump at *λ*_2_. A variable optical attenuator (VOA1) is inserted to balance the pulse peak power at the two wavelengths. On the other hand, the lower branch experiences the carrier-suppressed modulation at a frequency around Brillouin frequency shift (BFS) to generate the double sideband probe signals at the two wavelengths. The carrier suppression ratios for both wavelengths are measured to be over 30 dB. The EDFA in the lower branch are employed to control the power of the probes. Another circulator and FBG2 are used to separate the probe signals at *λ*_1_ and *λ*_2_ as two channels of the BD-BOTDA system. Similar to the pump branch, BPF3 and VOA2 play the same role as BPF2 and VOA1. Then, in each direction, a 50/50 coupler is utilized to combine a pulsed pump at *λ*_1_ (or *λ*_2_) together with a CW probe at *λ*_2_ (or *λ*_1_), and the outputs of the two couplers are delivered to the FUT from both ends. At the receiver side, the probe signals at *λ*_1_ and *λ*_2_ are amplified by EDFAs, and the corresponding lower sideband signals are selected by using circulators and FBGs (FBG3 and FBG4) for detection. The 3 dB bandwidth of the FBG3 and FBG4 is ~10 GHz. The selected signals at *λ*_1_ and *λ*_2_ are detected by two photodetectors (PD1 and PD2, Newport Corporation, Irvine, CA, USA) simultaneously and then collected by using a two-channel oscilloscope (OSC, Tektronix China Ltd., Shanghai, China).

The FUT is composed of three spans of single-mode fibers (Sumitomo Electric Lightwave Corp., Raleigh, NC, USA): FUT I (40.6 km), FUT II (1 km), and FUT III (41.3 km), as shown in Figure 2. FUT II is placed at the middle of the other two. Hence, FUT I is measured with the *λ*_1_ channel, while FUT III is measured with the *λ*_2_ channel at the same time. The last 2 m fibers of FUT I and FUT III are cooled down to 0 °C by using an ice-water bath, while the remaining fibers are kept under room temperature (about 24 °C).

During the experiment, the frequency is scanned from 10.73 to 10.93 GHz and 1000 averaging times are adopted on the oscilloscope to minimize the noise. Note that the BFS is related to the wavelength of the pulsed pump, thus in order to avoid the increase of frequency scanning range, the wavelength difference of the two channels of the BD-BOTDA system is chosen to be around ~3 nm, which only produces about 20 MHz BFS difference and avoids the interference between the two channels at the same time. At the same time, the 3 nm wavelength difference between the two channels also provides sufficient phase mismatch to minimize the effect of four-wave mixing (FWM) between the two channels. Moreover, only one pulse exists inside the fiber for each channel, which reduces the effective interaction length for FWM to take place.

To optimize the performance of each channel, the powers of the pump and probe signals have been carefully controlled. The extinction ratio of the pump pulses at both wavelengths is measured to be over 40 dB, and the peak power, limited by modulation instability (MI) [5], is set to be 22 dBm, which is similar to that of a conventional unidirectional system. In comparison with the conventional setup, the probe lightwave in each channel will propagate through the carry-over fiber of about 40 km, and suffers about 8 dB attenuation before entering into its sensing fiber. Considering this fact, the average power of each probe lightwave launched into the FUT is ~2 dBm. By doing this, when it enters into the sensing half, the power is maintained at about −6 dBm, and the non-local effects can be avoided [3]. Under those power settings, the input powers of the pump and probe signals for sensing in each channel of the BD-BOTDA system are similar to those in conventional BOTDA system with a 40 km sensing range, resulting in the SNR performance of the BD-BOTDA system comparable to the conventional BOTDA system with half of the sensing range. Hence, with respect to 80 km sensing range, an SNR improvement of 8 dB can be expected using the approximated model in [20] when compared with the conventional system of the same sensing range. It should be noted that we have monitored the optical spectrum of the signals from both fiber ends and have not observed any FWM phenomena at the power levels used in our experiment.

## 3. Experiment Results and Analysis

**Figure 3:**
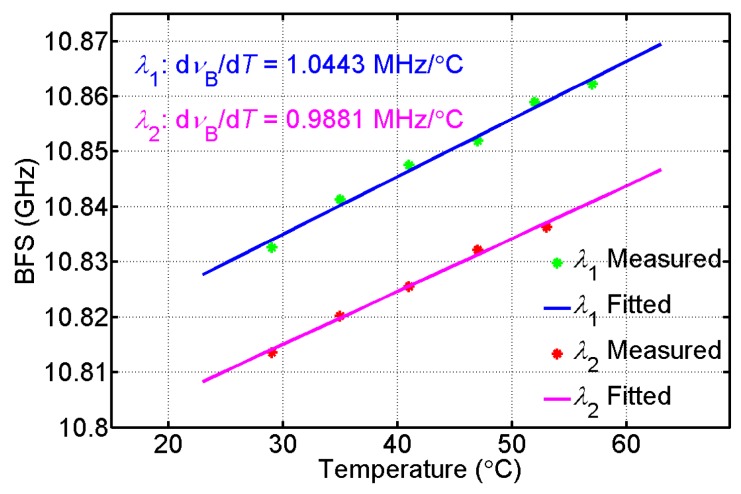
Brillouin frequency shift versus temperature at two wavelengths: *λ*_1_ and *λ*_2_.

Before we start the use of the BD-BOTDA system for sensing, we first calibrate the temperature coefficients of BFS at both wavelengths *λ*_1_ and *λ*_2_. The results are given in Figure 3, showing linear relationships between BFS and temperature. The temperature coefficients at *λ*_1_ and *λ*_2_ are 1.0443 and 0.9881 MHz/°C, respectively, which will be used in the calculation of temperature distribution along the FUT.

**Figure 4:**
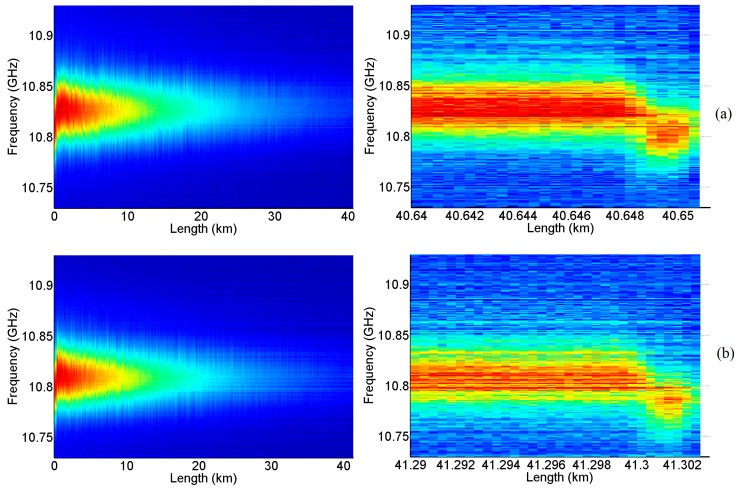
BGS as a function of distance measured at (**a**) the *λ*_1_ channel along FUT I (left) and the corresponding zoom-in view of the last 10 m of the fiber (right), and (**b**) the *λ*_2_ channel along FUT III (left) and the corresponding zoom-in view of the last 12 m of the fiber (right).

Then, we apply the BD-BOTDA system for distributed temperature sensing of the FUT. Figure 4 shows the BGS distribution versus distance for both the *λ*_1_ and *λ*_2_ channels. As mentioned above, each channel is responsible for the measurement of one half of the FUT, and Figure 4a shows the distributed BGS measured at the *λ*_1_ channel along FUT I and FUT II, while Figure 4b plots the BGS distribution measured at the *λ*_2_ channel along FUT III. For both channels, the BGS distributions along the last ~10 m of FUT I and FUT III are also illustrated at the right side of Figure 4, showing clearly the cooled 2 m fibers in FUT I and FUT III.

**Figure 5:**
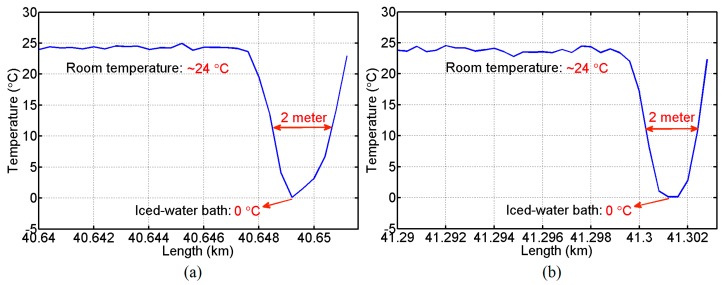
Temperature distribution measured with (**a**) the *λ*_1_ channel at the far end of its sensing fiber and (**b**) the *λ*_2_ channel at the far end of its sensing fiber.

By using the temperature coefficients calibrated in Figure 3, we obtain the temperature distribution along the FUT from the measurement of the two channels of the BD-BOTDA system. Figure 5a,b show the corresponding temperature information for the final meters of FUT I and FUT III. The fiber section cooled down to 0 °C has been successfully detected by each wavelength channel. The results also indicate a spatial resolution of ~2 m (corresponding to 20 ns pump pulses used in our experiment) in both channels of our system.

## 4. Discussion

**Figure 6:**
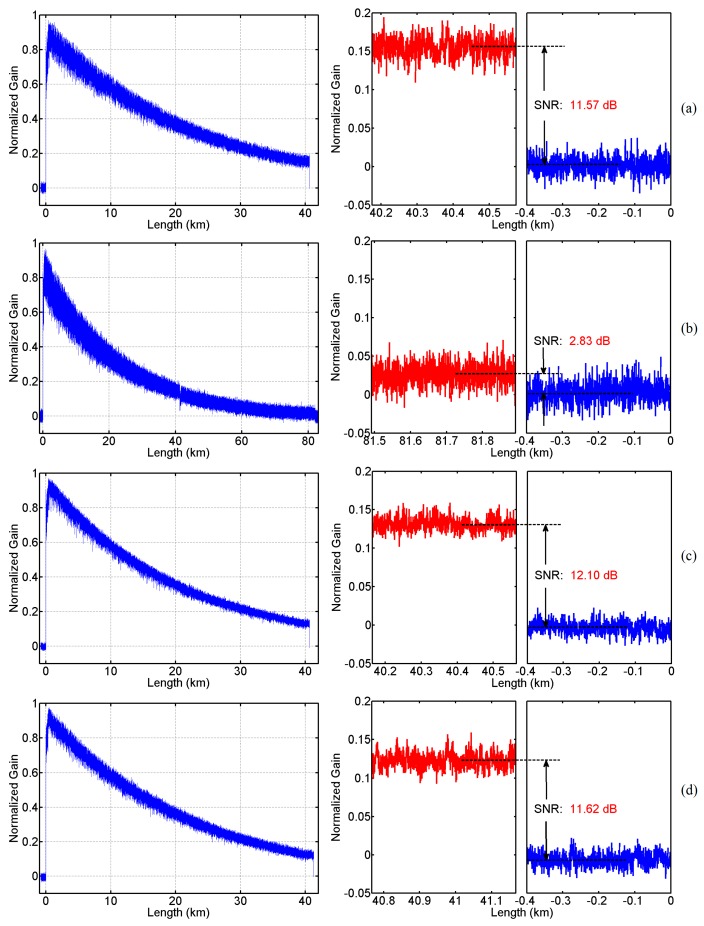
Normalized Brillouin time domain trace of (**a**) the 40.6 km SMF measured with the unidirectional BOTDA system (left); (**b**) the 81.9 km SMF measured with the unidirectional BOTDA system (left); (**c**) the λ_1_ channel measured with the BD-BOTDA system (left); and (**d**) the λ_2_ channel measured with the BD-BOTDA system (left). Each trace is measured at Brillouin center frequency shift of the SMF. Insets: the contrast between the Brillouin signal near the end of the fibers (red curve) and the noise background (blue curve) on the right hand side.

In order to illustrate the advantages of our BD-BOTDA system, two SMFs of 40.6 km and 81.9 km have been measured for comparison respectively by using a unidirectional BOTDA system with almost the same parameters as those in the BD-BOTDA system. The Brillouin time-domain traces measured at the Brillouin center frequency of the SMFs are given in Figure 6. Figure 6a,b show the traces along 40.6 km and 81.9 km SMFs measured with the unidirectional BOTDA system, while Figure 6c,d depict the traces of the λ_1_ and λ_2_ channels in the BD-BOTDA system, respectively. The four insets at the right side of Figure 6 illustrate the contrast between the Brillouin signal (red curves) near the end of the fibers and the noise background (blue curves), from which the SNRs of the traces are calculated to be 11.57 dB, 2.83 dB, 12.10 dB, and 11.62 dB. The SNRs of the two channels in the BD-BOTDA system are almost the same as the trace SNR of the 40.6 km fiber measured with the unidirectional BOTDA, and have an SNR enhancement of over 8 dB when compared to the unidirectional BOTDA system with a sensing range of ~81.9 km. The enhanced performance is also verified by calculating the BFS distribution along the fiber.

According to the results and Figure 6 shown above, the proposed technique shall offer a doubled sensing range while maintain the SNR at the level of the conventional BOTDA with half of the sensing distance. Moreover, when compared to the conventional BOTDA with the same FUT range, an SNR enhancement corresponding to the attenuation of the half FUT length, which is ~8 dB in our demonstration, can be achieved by employing the BD-BOTDA system.

**Figure 7:**
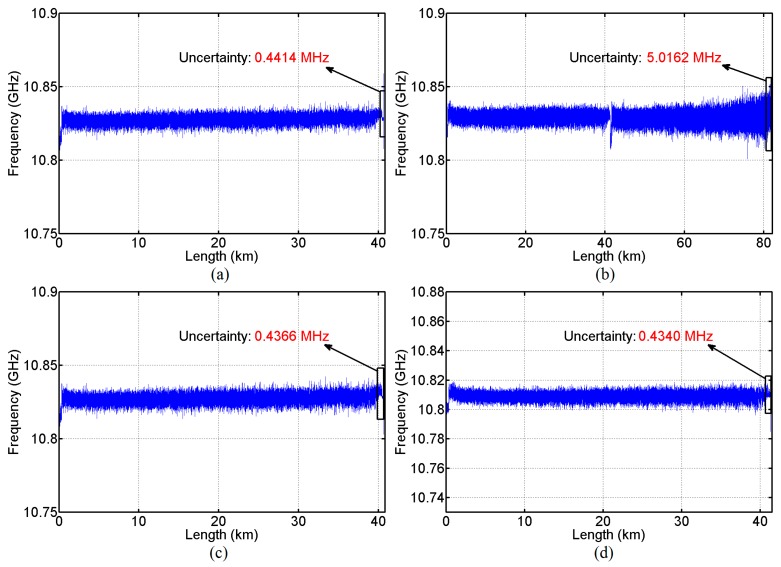
BFS versus distance of (**a**) the 40.6 km SMF in the unidirectional BOTDA system; (**b**) the 81.9 km SMF in the unidirectional BOTDA system; (**c**) the *λ*_1_ channel in the BD-BOTDA system along FUT I; and (**d**) the *λ*_2_ channel in the BD-BOTDA system along FUT III.

By using Lorentzian curve fitting, the BFS distributions for the 40.6 km and 81.9 km SMFs measured with the unidirectional BOTDA are shown in Figure 7a,b for comparison. The results measured with the *λ*_1_ and *λ*_2_ channels of the BD-BOTDA system are shown in Figure 7c,d. The BFS uncertainties of the *λ*_1_ and *λ*_2_ channels in the BD-BOTDA system are calculated to be 0.4366 MHz and 0.4340 MHz, respectively; the ones in the unidirectional BOTDA system for 40.6 km and 81.9 km SMFs are 0.4414 MHz and 5.0162 MHz. Obviously, the BFS uncertainty of our BD-BOTDA system with a sensing range of 82.9 km is comparable to that of the unidirectional BOTDA system with half of the sensing range, and hence is much better than that of the unidirectional BOTDA system with a similar sensing range. It is worth mentioning that the BFS uncertainty of the 81.9 km SMF using the unidirectional BOTDA system suffers from a degradation of over 10 dB, rather than the expected ~8 dB degradation, compared to the case of the 40.6 km SMF. This may be because the BGS profile near the end of the 81.9 km SMF has been flattened and broadened. Note that the undistorted BGS’s measured with the BD-BOTDA system indicate that the non-local effect is negligible in our experiment thanks to the power optimization.

Based on the analysis above, the proposed technique doubles the sensing range while maintaining the same performance as a conventional BOTDA with half of the range. Moreover, it is worth mentioning that the proposed technique shows its benefits for the practical deployment of the BOTDA system. The loop configuration for most of the BOTDA systems, in which the FUT goes and comes back by the same path, makes the second half redundant for monitoring. Since the proposed BD-BOTDA technique offers the same level of performance for each half of the FUT, the redundant data collected from one half of the FUT can serve as an appropriate reference for the discrimination of temperature and strain. Therefore, by carefully deploying the FUT making half of it free from strain as reference and the other half experiencing both environmental temperature and strain, the configuration redundancy in the BD-BOTDA system can be properly used for distributed monitoring of both temperature and strain.

## 5. Conclusions

In summary, a novel scheme of the BD-BOTDA system has been proposed and experimentally demonstrated by using two pump-probe pairs at two different wavelengths with each channel measuring one half of the fiber. Based on this scheme, distributed temperature sensing of up to 82.9 km sensing range at a spatial resolution of 2 m has been realized without any complicated configurations and extra amplifications. Compared with the unidirectional BOTDA technique of a similar sensing range, our scheme has enhanced the SNR by over 8 dB and achieved the BFS uncertainty of ~0.44 MHz. Better performance with a longer sensing range can be expected if the parameters of the two channels are further optimized and balanced. We believe the BD-BOTDA technique can be easily applied together with the existing BOTDA techniques for further enhancement of the sensing performance.
